# Plant cold acclimation and its impact on sensitivity of carbohydrate metabolism

**DOI:** 10.1038/s41540-025-00505-1

**Published:** 2025-03-19

**Authors:** Stephan O. Adler, Anastasia Kitashova, Ana Bulović, Thomas Nägele, Edda Klipp

**Affiliations:** 1 Theoretical Biophysics, Institute of Biology, Humboldt-Universität zu Berlin, Berlin, Germany; 2 Plant Evolutionary Cell Biology, Faculty of Biology, Ludwig-Maximilians-Universität München, Planegg-Martinsried, München, Germany

**Keywords:** Computational biology and bioinformatics, Plant sciences, Systems biology

## Abstract

The ability to acclimate to changing environmental conditions is essential for the fitness and survival of plants. Not only are seasonal differences challenging for plants growing in different habitats but, facing climate change, the likelihood of encountering extreme weather events increases. Previous studies of acclimation processes of *Arabidopsis thaliana* to changes in temperature and light conditions have revealed a multigenic trait comprising and affecting multiple layers of molecular organization. Here, a combination of experimental and computational methods was applied to study the effects of changing light intensities during cold acclimation on the central carbohydrate metabolism of *Arabidopsis thaliana* leaf tissue. Mathematical modeling, simulation and sensitivity analysis suggested an important role of hexose phosphate balance for stabilization of photosynthetic CO_2_ fixation. Experimental validation revealed a profound effect of temperature on the sensitivity of carbohydrate metabolism.

## Introduction

Dynamic environmental conditions affect plant metabolism, growth, and development. Due to their sessile lifestyle, plants have developed mechanisms to respond to changing environmental conditions, e.g., elevated light intensities, low temperatures, or drought. The reversible adjustment of metabolism to such factors is termed acclimation and comprises various responses on a molecular and physiological level^1^.

Acclimation has been studied in diverse plant species, e.g., *Arabidopsis thaliana*^2^, *Zea mays*^3,4^ and *Nicotiana tabacum*^5^. When exposed to temperature changes, besides the need for protection from freezing or adverse heat, photosynthesis and metabolism needs to be stabilized immediately to prevent irreversible tissue damage.

Because the velocity and balance of involved chemical reactions is differently affected by temperature, these processes are likely to become unbalanced. This effect needs to be compensated. The compensation can happen actively, e.g., through changes in gene expression or molecular modifications, or passively, e.g. through the architecture of the reaction network and the physical properties of its components^6^. Besides having a compensatory nature, the reaction network can offer potential for regulation^7^. Taken together, these processes form a complex system of intertwined mechanisms that is difficult to entangle. Following approaches that combine different experimental procedures with mathematical modeling has shown to be a fruitful strategy to address complex problems like this.

During cold acclimation of *Arabidopsis thaliana*, a reprogramming of several metabolic processes has been described. These include the accumulation of soluble sugars, such as sucrose and raffinose, which potentially act as cryoprotectants and stabilize metabolism^2,8,9^. This is accompanied by the induction of *BAM3* expression^10–12^, an amylase that is involved in starch breakdown, leading to a decrease of starch amounts during the first hours of acclimation and supporting the accumulation of soluble sugars^13^. Later during acclimation, starch accumulates^8^. Additionally, the activity of TCA cycle enzymes and oxidative respiration is reduced by low temperature, resulting in a decrease of respiration and energy production if not counteracted and regulated^14^. Similarly, the activity of enzymes involved in photosynthesis are reduced in the cold, causing a decreased rate of carbon fixation and the accumulation of reducing equivalents, such as NADPH, which are used for biosynthetic processes^14–16^. Alongside these processes within the primary metabolism, also secondary metabolism is affected in its regulation. Under low temperature, flavonoids accumulate and it has been demonstrated that this accumulation process plays a central role in cold acclimation and freezing tolerance^17–20^. However, regulation at the interface of primary and flavonoid metabolism is only poorly understood.

Different genotypes of *Arabidopsis thaliana* exhibit different characteristics in their reprogramming of metabolism that are related to their sensitivity to cold. There are, for example, indications for differences of interconversion rates between hexoses and sucrose or of export rates to sink organs^21^. Mutants like *bam3*, *pgm1*, *chs* and *f3h* are deficient in processes directly involved in acclimation. The *bam3* mutant is deficient in amylase activity that is upregulated in cold conditions. This leads to insufficient retrieval of carbon from starch. The *pgm1* mutant, in contrast, is starch deficient because it lacks phosphoglucomutase that catalyzes an initial step of starch synthesis^22^. While these mutations are starch related and, therefore, correspond to primary metabolism the *chs* and *f3h* mutations are related to specialized metabolism and show deficient enzyme activities of the flavonoid biosynthesis pathway. Chalcone synthase (CHS) catalyzes the first step of flavonoid biosynthesis from malonyl-CoA and coumaroyl-CoA. In a later step, F3H and F3’H (flavanone 3-hydroxylase and flavanone 3’-hydroxylase) catalyze the conversion of naringenin, a precursor of a subgroup of flavonols including anthocyanins, into dihydroflavonol^23,24^.

Despite the numerous successful studies using different experimental and computational approaches, many aspects of the complex and dynamic mechanisms and processes during cold acclimation are still elusive. In an earlier study^25^ (Kitashova et al. 2022), the natural accession Columbia-0, together with the mentioned four mutants *bam3*, *pgm1*, *chs* and *f3h* were used to investigate regulatory interactions between primary and secondary metabolism during acclimation. Data on metabolite levels, enzyme activities and photosynthetic carbon uptake was obtained and used to calibrate a condensed mathematical model, comprising core elements of the central carbon metabolism of plant leaves. In this way, evidence for dynamic carbon partitioning between starch, sucrose and anthocyanin metabolism, including a unidirectional signaling link between starch and flavonoid metabolism and a central role of sucrose could be identified.

In the present study, we analyzed this model to investigate the effects of variations in light intensity on the five genotypes of *Arabidopsis thaliana* during and after two weeks of cold acclimation. We re-estimated the model parameters with literature-based constraints for coherent enzyme specific values across the different genotypes and investigated the sensitivity towards dynamics of net photosynthetic CO_2_ assimilation rates after different periods of high light exposure at 4 °C in vivo and in silico. A comparison of the dynamics showed a good agreement between experiments and simulations. This enabled for broader analysis of these dynamics through further simulations and the introduction of a sensitivity measure (sensitivity score). In combination with the analysis of control coefficients within the system, this approach indicates that temperature has a stronger impact on the overall sensitivity of central carbon metabolism than the different mutations and that there is likely one or more compensation or rescue mechanisms shared by primary and secondary metabolism.

## Results

In order to investigate the influence of dynamic CO_2_ assimilation rates on the acclimation process of the central carbon metabolism in *Arabidopsis thaliana* leaves, we investigated the model presented in the following section in a restricted parameter space. Therefore, we defined a procedure for assessing the dynamic sensitivity to these perturbations and compared its outcome when applied to model simulations or new experimental data. Additionally, we embedded the condensed model into a large-scale metabolic model and compared the resulting fluxes when constrained accordingly for further validation.

Our presented analysis, together with the evaluation of parameter control coefficients, could reveal an important role of hexose phosphate balance, a rearrangement of sensitivities in response to temperature change and potential common metabolic compensation mechanisms.

### The model

This work is based on the model presented before in Kitashova et al.^25^. In this study, different genotypes (wild type: Col-0; mutants: *chs*, *f3h*, *bam3*, *pgm1*) of *Arabidopsis thaliana* were exposed to 4 °C over the course of 14 days. On days 0, 1, 3, 7 and 14, samples were taken and net photosynthesis (nps), levels of starch, soluble carbohydrates, hexose phosphates, anthocyanins, and organic acids, as well as enzyme activities were quantified. The experimental data was then used to estimate parameters for simulation of the central carbon metabolism and its interface with flavonoid biosynthesis. Here, the parameters were optimized to meet a steady-state assumption for each genotype and time point pair, as described in the methods section, resulting in 25 conditional model realizations (parameterizations) that represent different stages of acclimation for the various genotypes.

**Fig. 1:**
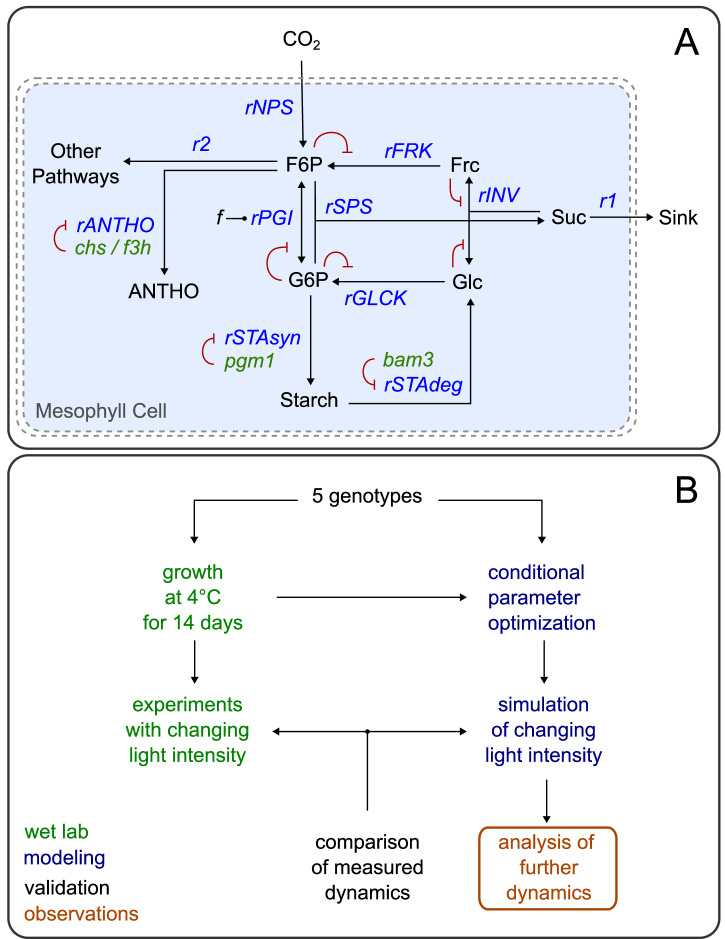
Model scheme and workflow. **A** The model consists of 5 main metabolites: F6P, G6P, Frc, Glc and Suc. These metabolites take part in various enzymatic reactions (*rSPS*, *rPGI*, *rFRCK*, *rGLCK*, *rINV*) or biological processes (*rNPS*, *rSTAsyn*, *rSTAdeg*, *rANTHO*). These are depicted as arrows. There are two export fluxes, r1 to sink organs and r2 to other pathways of the central carbon metabolism in the mesophyll cell. The green elements (*chs/f3h, pgm1, bam3*) represent the 4 different mutations and how they affect the reaction network. Red lines indicate either negative feedback regulation by metabolites on reaction fluxes or negative effects of mutations on enzymatic reactions. Additionally, the parameter f is shown, which balances the *rPGI* reaction, as indicated in the rate equations. **B** This flowchart provides an overview on the workflow and steps in this study. It highlights the experimental (green) and modeling (blue) part and how these are connected. Observations and validation are represented in orange and black, respectively.

The basic model comprises 5 metabolites and 12 reactions and is described by a set of ordinary differential equations (ODEs) given in Equation System 1 and a set of corresponding rate equations (Equation System 2). A scheme of the model is shown in Fig. 1.

In the model, fructose 6-phosphate (F6P) is assumed to be the main product of photosynthesis available for further processes. It is in an equilibrium with glucose 6-phosphate (G6P), that is defined by phosphoglucoisomerase (PGI) activity. F6P and G6P together can be converted into sucrose (Suc) by sucrose phosphate synthase (SPS), while only a fraction of G6P is available for this reaction. Here, UDP-glucose as an intermediate metabolite was omitted for simplification, with the assumption that about 20-50% of G6P are converted before entering the SPS reaction. Suc is then directly exported to sink organs. Additionally, F6P is converted into anthocyanins (ANTHO) and diverted to the second export flux (r2) while G6P is converted into starch in a single process (rSTAsyn) lumping the stepwise synthesis. The invertase reaction (rINV), breaking down Suc into fructose (Frc) and glucose (Glc), together with the fructokinase and glucokinase reactions (rFRCK, rGLCK), phosphorylating Frc and Glc and resulting in the recovery of F6P and G6P, form a central loop that enables responsive balancing of metabolites.

### Amounts of F6P and G6P are differentially affected by mutations in flavonoid and starch metabolism

**Fig. 2:**
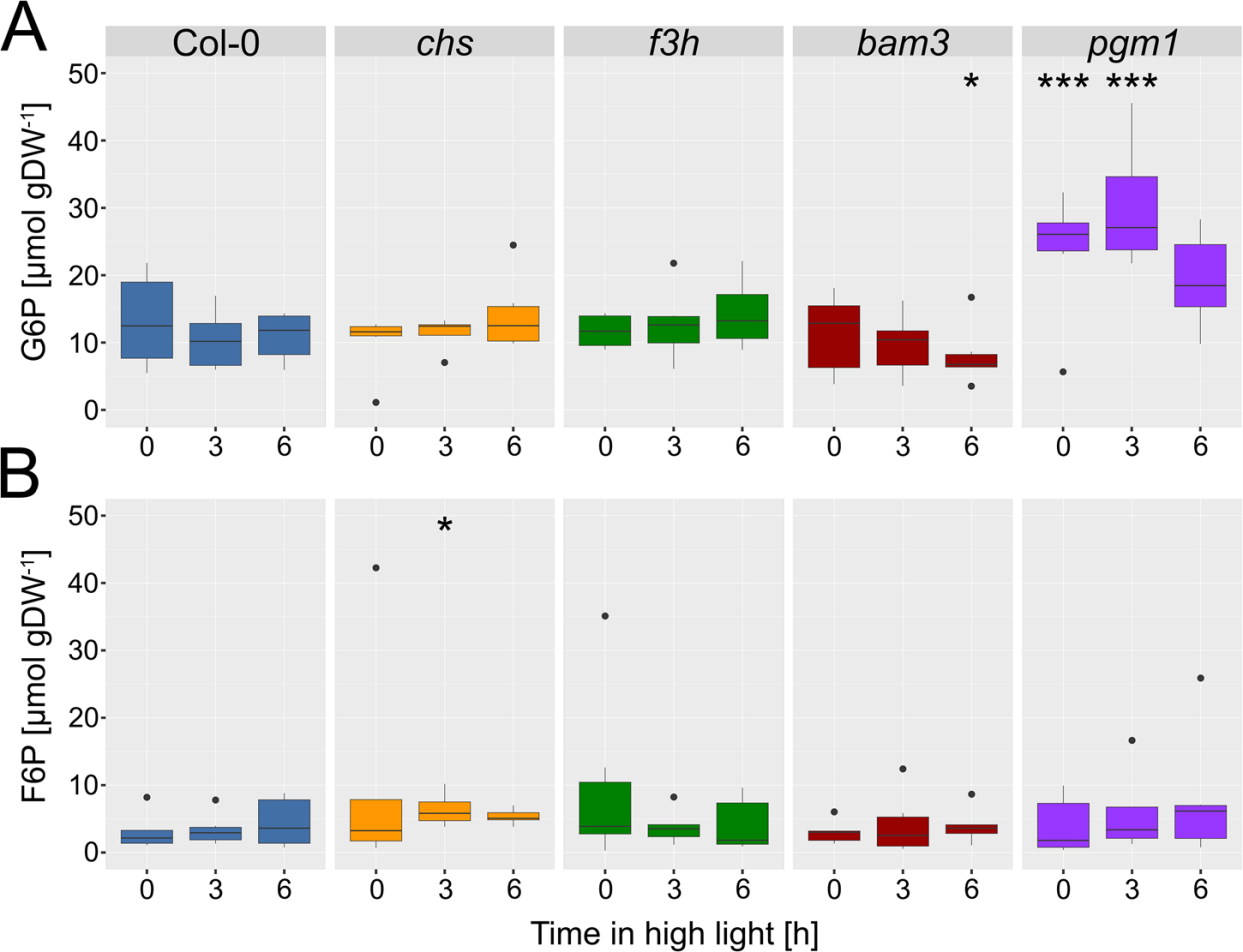
Hexose phosphate dynamics under cold and high light. **A** Dynamics of glucose 6-phosphate (G6P) in Col-0 (blue), *chs* (orange), *f3h* (green), *bam3* (red), and *pgm1* (purple). **B** Dynamics of frucose 6-phosphate (G6P) in Col-0 (blue), *chs* (orange), *f3h* (green), *bam3* (red), and *pgm1* (purple). Asterisks indicate significance compared to amounts in Col-0 (ANOVA, *p < 0.05, ***p < 0.001; *n* = 4–6).

To investigate the effects of cold acclimation on sensitivity towards changes in light intensity and assess our models performance in this regard, we quantified hexose phosphate amounts in cold acclimated plants at 4 °C before and after 3 h and 6 h exposure to elevated light (Fig. 2). While the median values of F6P ranged from 2 to 6 µmol gDW^−1^ those of G6P ranged up to 26 µmol gDW^−1^, the value distributions of the single measurements shared roughly the same range between 0 and 45 µmol gDW^−1^ (Fig. 2). In *chs*, the amount of F6P tended to increase after 3 h of high light while this effect was not significant (Fig. 2B). Both *pgm1* and *bam3* mutants were significantly affected in G6P metabolism, and *pgm1* accumulated highest amounts across all genotypes. However, between 3 h and 6 h of high light exposure, G6P amounts decreased again in plants of both *pgm1* and *bam3* which was not observed in Col-0, *chs* and *f3h*. (Fig. 2A).

### Model validation

**Fig. 3:**
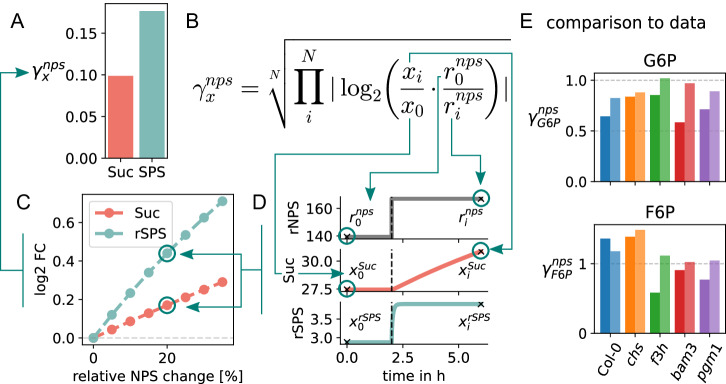
Estimation of sensitivity scores *γ*_x_^nps^ and comparison to experimental data. **A** Sensitivity scores of sucrose concentration (Suc) and the SPS reaction flux (rSPS) of model Col-0 day 0. **B** Formula for the sensitivity score *γ*_x_^nps^. **C** Log_2_ fold-changes of Suc and SPS for computed net photosynthesis (nps) increases. **D** Exemplary time courses of underlying simulations for a change in nps rate (rNPS) of 20%. Shown are the time courses of rNPS, Suc and rSPS. The vertical black lines indicate the time point of rNPS increase (2 h). The “x”s mark the corresponding values for the computation of *γ*_x_^nps^. **E** Sensitivity scores computed from control experiments (darker bars) are compared to sensitivity scores of simulations (lighter bars).

To assess the impact of acclimation on central carbon metabolism in model simulations, the different conditional model realizations were subjected to external perturbations, namely changes in net photosynthesis (nps) rates. Under in vivo conditions, variations in nps result from naturally changing light. Starting from steady states that were derived from the experimental data, the nps rates in the model were increased or decreased by 5 to 30% in 5% increments and 4 h time courses were simulated. The resulting changes in metabolite levels and reaction fluxes were then taken together to compute the sensitivity of the respective condition to these perturbations as

1 $${{\rm{\gamma }}}_{{\rm{x}}}^{{\rm{nps}}}={\root{N}\of{\mathop{\prod }\limits_{i}^{N}\left|{lo}{g}_{2}\left(\frac{{x}_{i}}{{x}_{0}}\cdot \frac{{r}_{0}^{{nps}}}{{r}_{i}^{{nps}}}\right)\right|}}$$

where *γ*_x_^nps^ is the sensitivity score of measure x (metabolite concentration or flux) on perturbations of nps, N is the number of performed perturbations (increase/decrease, 5%, 10%, …), x_0_ is the initial steady state value of measure x and x_i_ the resulting value after perturbation i. The ratio of initial nps rates and rates of perturbation i are represented by r_0_^nps^ and r_i_^nps^, respectively. Figure 3 illustrates this procedure and provides a graphic representation of how well the results match those derived from the experimental data.

To evaluate the suitability of the described analysis of sensitivity to variations in nps rates, we compared *γ*_x_^nps^ derived from control experiments to values estimated from results of model simulations mimicking the experimental procedure of a newly performed experiment to specifically test the sensitivity in vivo. To this end, *Arabidopsis thaliana* plants from all 5 genotypes were grown at 22°C and transferred to 4 °C for 14 days for cold acclimation. On day 14, the plants were either exposed to normal light (100 µmol m^−2^ s^−1^) and sampled after 3 h, or plants were transferred to elevated light (240–270 µmol m^−2^ s^−1^) and samples were taken after 3 and 6 h. While, in the present study, nps rates were not determined experimentally, we have shown earlier that an increase of light intensities at 4 °C from 100 to 250 µmol m^−2^ s^−1^ results in an increase of nps rates by 20–30%^26^. Hence, the shift to high light was simulated by an increase of rNPS by 25%. From the obtained experimental and simulation data *γ*_x_^nps^ values were computed according to Eq. 1 with the simple adjustment that, instead of different perturbation intensities, x_i_ represent different durations of exposure (3 h and 6 h). The concentrations obtained under normal light are the reference values x_0_ and r_0_^nps^. This comparison is shown in Fig. 3E. We see a very good agreement between experimentally obtained values and simulation results. For each metabolite there is one genotype (*bam3* for G6P and *f3h* for F6P) where the model predictions deviate more than 50% from the data. In summary, the overall agreement verifies a good suitability of the presented sensitivity assessment.

To further validate the model and test its performance, especially considering the degree of condensation, we compared it to a large(r)-scale metabolic model.

Because the tackled phenomenon of carbon partitioning into starch and sucrose involves some highly connected metabolites (used in many reactions), we wanted to make sure that our model simplification still fits reasonably well into the broader context of the metabolic network of *A. thaliana*. For this, we compared the flux distribution from the dynamic model proposed here, and the one obtained through a Flux Balance Analysis (FBA)^27^ simulation of a genome-scale metabolic reconstruction of *A. thaliana* known as AraCore^28^.

All comparisons were done for the Col-0 genotype, as the other strains involve either interruptions in the pathways, used to produce biomass precursors (which make the strains non-viable in FBA context), or reduce the efficiency of a cellular process, a subtlety which cannot be considered in a stoichiometry-based modeling framework such as FBA.

We used several fluxes from the dynamic model to constrain the AraCore model. The import reaction to F6P was used to constrain the flux through FBPase (both cytosolic and chloroplastic), the major contributor to synthesis of F6P through the Calvin-Benson-Bassham cycle. Additionally, we used the dynamic model fluxes to constrain the fluxes through S6P synthase (S6PS_c), G6P isomerase (PGI_c), and hexokinase (HEXK_c). The lower and upper bound of those reactions were set to 90 and 110% of the flux values obtained in the dynamic model.

We then compared (1) the flux *r2* (flux going out from F6P, see Fig. 1) to the flux through F6P transketolase which is a major pathway through which F6P is used for replenishing of the Calvin-Benson-Bassham cycle intermediates, (2) the total flux going out of sucrose in both our dynamic model and the FBA model and (3) the total rate of starch synthesis. One outgoing flux from F6P, which we did not compare to the AraCore prediction, is the production of anthocyanins. The reason for this is that anthocyanins are not explicitly modeled in the condensed dynamic model, and their modeled precursor, shikimate, is not exclusively used for anthocyanin synthesis. Due to this discrepancy, we decided that this comparison would not be meaningful.

**Fig. 4:**
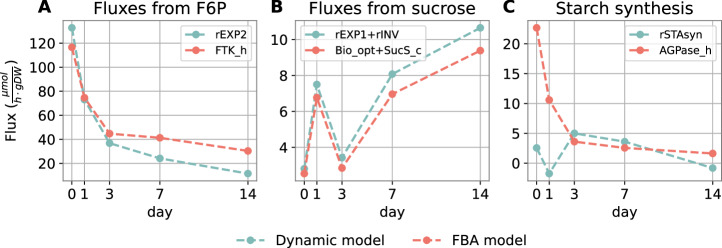
Comparison of flux values between the dynamic model and FBA. **A** Comparison between *r2* dynamic model flux and the flux through FTK reaction in the chloroplast facilitating replenishment of CBC metabolites. **B** Comparison of outgoing fluxes of sucrose (invertase and export flux *r1*). **C** Comparison between starch synthesis fluxes.

When analyzing the results in detail, we see that the major flux depleting F6P (*r2* in dynamic model) matches well (in order of magnitude and exact quantitative value) to the one predicted by AraCore (see Fig. 4A). Since these are some of the fluxes with highest absolute values, this indicates that the major stoichiometric constraints on the network are well represented in the dynamic model, and that it can reliably be used as a proxy for the global metabolic state of the modeled metabolites.

Next, the total fluxes going out of sucrose match almost exactly in both models (see Fig. 4B). However, we have to point out that the values of two individual fluxes that go out of sucrose in the dynamic model, i.e. the export flux *r1* and the flux through the invertase, do not match well to their corresponding fluxes in the FBA model. These are not shown separately in Fig. 4, but as a combined flux to illustrate the overall fluxes away from sucrose in both models. The predicted export flux of sucrose is much smaller in the FBA model due to the fact that it is represented directly by the biomass reaction. Since the biomass reaction requires many metabolites in a particular stoichiometric relation, the flux through it cannot be easily changed, and is determined not only by sucrose, but by the entire state of the metabolic network. The comparison of starch synthesis does not match as well as the other two comparisons, even if the order of magnitude is not far off, especially after day 3 (see Fig. 4C). We assume this is because of the way in which the starch synthesis rate was initially calculated from starch measurements, by averaging the difference between starch quantity in the morning between consecutive days over 24 h (net balance).

Overall, we see a good agreement between the fluxes concerning the modeled metabolites in the dynamic and in the FBA model, giving further confidence in the developed model, the measurements used to calibrate it, and the calibration itself. On this basis, we will subject the model to a thorough analysis of parameter sensitivity in order to understand the role of individual parameters in the acclimation ability of the plants.

### Observations and insights

After investigating the plausibility of the model and parameters, first by comparing the sensitivity to variation of nps rates with new in vivo data and, second, by comparing the fluxes of our small dynamic model to an established genome-scale FBA model, we also did a classical sensitivity analysis by computing all flux, concentration, and parameter control coefficients to assess the impact of parameter choices on the model behavior. We used the control coefficients in the definition according to

2 $${{\rm{C}}}_{{\rm{p}}}^{{\rm{X}}}=\frac{{\rm{dln}}({\rm{X}})}{{\rm{dln}}({\rm{p}})}$$

where C^X^_p_ is the control coefficient of flux, concentration or parameter *p* over measure *X* (flux or concentration)^29,30^. It describes how strong a variation of *p* affects the steady state values of *X*. In this sense, it differs from the approach described before, because it always considers a relaxed system.

The results of this systematic sensitivity analysis are described in Supplementary Section 1 and illustrated in Supplementary Figs. 1 and 2. In general, we see a variable influence of most parameters, depending on the given conditions, while no clear trend is apparent. For some parameters, the control coefficients show 2 modes, representing conditions under which the given parameter has an influence or doesn’t have an influence (e.g., due to substrate depletion). The same observations hold for fluxes and concentrations.

Notably, the parameter control coefficient of *f*, the parameter that determines the balance between F6P and G6P at the PGI reaction was significantly (4–5 times) larger than all other control coefficients. An overview of this analysis is provided in Supplementary Section 1.

To identify critical parameters and conditions, control coefficients with values larger than 1.5 were investigated more closely. This is the point at which the distribution of control coefficients exhibits a clear drop and a relatively wide tail, meaning that most values are smaller, but a few are considerably larger than 1.5. Interestingly, these values could not clearly be attributed to certain days or genotypes during acclimation, but largely to fructose, glucose and reactions connected to them. This suggests a comparably high sensitivity of this part of central carbon metabolism to perturbations within the system. Since this lack of dependence on genotype or duration of cold exposure was an unexpected result, we decided to investigate whether there might be a sensitivity dependence on temperature instead.

**Fig. 5:**
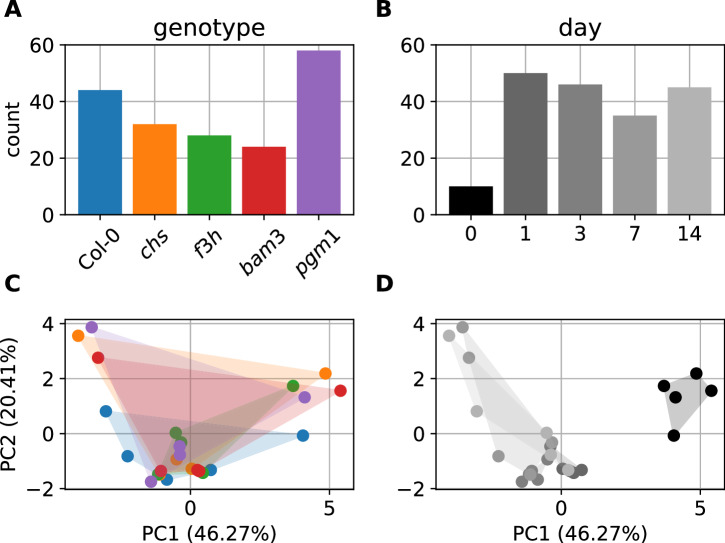
Comparison of model features by genotype or day. The upper panel shows the frequency of parameter control coefficients larger than 1.5 for each genotype (**A**) or day (**B**). The lower panel shows a two-component PCA for the variations in *γ*_x_^nps^. The color attribution of the dots in the lower panels correspond to those in the upper panel (**A** → **C,** **B** → **D**). The shaded areas in the PCA plots serve a better identifiability of related dots.

The investigation of high value control coefficients revealed two additional indications. First, when comparing the frequency of these values between genotypes the values increase from *bam3* along the flavonoid mutants to Col-0 arriving at *pgm1* with the highest frequency. Thus, defects in either starch synthesis or degradation seemed to have opposite effects on this centralization of increased sensitivities around Frc and Glc. Second, when comparing the frequency of high value control coefficients between days, day 0 significantly stands out, having a much smaller frequency (10 compared to 35–50). This means that the described sensitivities around Frc and Glc are particular to low temperatures, as they clearly become more pronounced during and after acclimation to 4 °C. Both of these observations are illustrated in Fig. 5A, B.

To investigate differences in nps variation responses between different genotypes or throughout the course of acclimation, a principal component analysis on *γ*_x_^nps^ was performed. The two components with the highest contribution make up 46.27% (PC1) and 20.41% (PC2) of the differences between the values of all model realizations. The composition of these two components is given in Table 1. The analysis did not show any apparent trend when comparing different genotypes, but interestingly, it exposed an observation that goes along with the described investigation of high value control coefficients. When comparing the days, day 0 is significantly shifted along the PC1 axis, compared to all other days, as illustrated in Fig. 5C, D. This means that the variation of nps rates affects the system in a considerably different manner before and during or after the acclimation process. Since PC1 and PC2, together, account for 66.68% of the variation there are still components PC3 and PC4 adding a considerable amount to the variation with 14.72% and 11.77%, respectively. These components were also investigated, but no clusters or other particularities could be observed. A visualization of these components is provided in the Supplementary Fig. 3.

**Table 1:** PCA component composition

	PC 1	PC 2
F6P	−0.27	0.41
G6P	−0.27	0.04
Frc	−0.31	−0.24
Glc	−0.32	−0.19
Suc	−0.32	−0.32
rSPS	0.06	−0.08
rPGI	0.04	−0.18
rFRCK	−0.35	0.22
rGLCK	0.13	0.18
r1	−0.32	−0.32
rINV	−0.29	−0.28
r2	−0.27	0.41
rSTAsyn	−0.27	0.04
rANTHO	−0.27	0.41

**Fig. 6:**
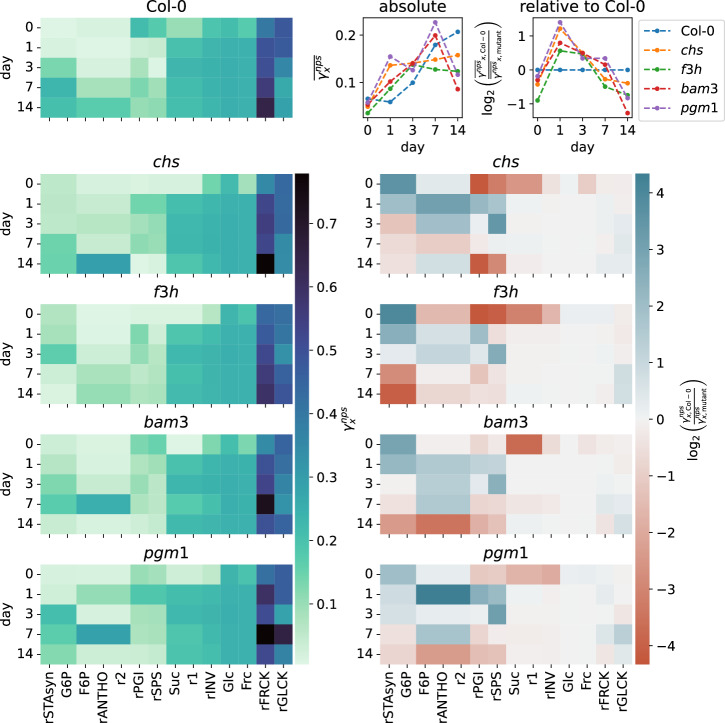
Overview of *γ*_x_^nps^ values. The heatmaps on the left side show the *γ*_x_^nps^ values for every model realization. For each genotype the evolution of values can be followed over days from top to bottom. Each column represents a different measure of the model, that is either a metabolite concentration or a flux. They are not ordered by type, but in the sense of related blocks within the model structure. The heatmaps on the right side show log_2_ fold-changes of these values when compared to the corresponding value of Col-0. The panel in the top right corner illustrates the evolution of the geometric mean of all *γ*_x_^nps^ values per day for each genotype. The left panel shows the absolute values, while the right panel, again, shows the log_2_ fold-changes compared to Col-0.

An overview of *γ*_x_^nps^ values for all genotypes and days is given in Fig. 6. We see that the rFRCK and rGLCK reactions are particularly sensitive to nps rate variations, and also the concentrations of Frc and Glc have relatively high values over all genotypes. Alongside an increase of rFRCK sensitivity over the course of acclimation, also those of F6P, anthocyanin synthesis and r2 increase. While peaking on day 14 for Col-0 and the flavonoid deficient mutants (*chs* and *f3h*), the peak already occurs on day 7 for the mutants with defects in starch metabolism (*bam3* and *pgm1*). Interestingly, the sensitivities of rFRCK, rGLCK, Frc, Glc are prominent compared to values of other measures, but are in a close range over all genotypes. This becomes evident when looking at the heatmaps in the right part of Fig. 6 depicting the log_2_ fold changes of all *γ*_x_^nps^ values compared to the corresponding values of Col-0. The fold changes of rFRCK, rGLCK, Frc, and Glc are always close to zero for all mutants.

Examining the other measures, we see a set of divergence patterns that appear to be conserved over all considered mutants. Clearly, there are apparent quantitative differences, but the qualitative behavior is fairly robust. The fold change for G6P and starch synthesis (rSTAsyn) starts at positive values and constantly declines, switching to negative values around day 3 for all mutants. Values of F6P, anthocyanin synthesis (rANTHO) and r2 start from a low or slightly negative fold change, switch to positive values during acclimation and return to a low or negative value after acclimation or, in the case of *chs*, already at day 7. The reaction fluxes rPGI and rSPS follow a similar pattern but return to low or negative values already at day 7 for all mutants. Here, the flavonoid mutants both show more negative values on day 0 than the starch related mutants. Suc, r1 and rINV start with negative fold changes that vanish during and after acclimation.

When following the mean *γ*_x_^nps^ values for each genotype over the course of days, as illustrated in the top right corner of Fig. 6, one can notice an overall increase of mean absolute sensitivity from day 0 to day 14. Considering Col-0 as the baseline sensitivity, we again see a similar pattern for all mutants, that is comparable to the behavior of PGI and SPS with a stronger decline at the end, due to the contributions of G6P and starch synthesis.

It is difficult to interpret these observations in terms of defined mechanistic insight. However, the emergence of similar divergence patterns from the wild type indicates that common rescue mechanisms might compensate for the deficiencies of these mutants, even if different parts of the central carbon metabolism are affected.

## Discussion

In this study we present detailed analysis of a condensed dynamic model of the core central carbon metabolism of *Arabidopsis thaliana* leaves. This model has previously been used to identify a role of limitations in sucrose synthesis in carbon partitioning during acclimation (Kitashova et al. 2022)^25^. Since it has been shown that light intensity is of great importance for the cold acclimation process of plants, and there are complex interactions between cold and light signaling^31^, a dynamic sensitivity analysis was defined, and new experiments were performed to assess its applicability. Also, the model has been refined with additional parameter constraints, according to literature^32–36^. Certainly, there are different strategies to calibrate dynamic models. We applied a steady state assumption, because we were considering long term changes (days compared to minutes or hours) and there were measures estimated as net balance from the experimental data (starch synthesis) for which this assumption is suitable. In terms of parameter estimation, we tried various procedures. These were: (a) finding the best optimization score for each condition individually without further restrictions, (b) for each condition individually with the restrictions explained in the methods section, (c) for conditions sharing the exact same K_m_/K_i_ values, (d) for each day in a pairwise K_m_/K_i_ fitting procedure. While (c) did not result in any successful optimization, parameter sets were found for (a), (b) and (d). Their performance in comparison to the experimental data was tested and (b) scored the best. Since it also follows reasonable restrictions for the kinetic parameters derived from literature, we are confident that it is best suitable and provides the most reliable predictive power for the presented study. Additionally, it has been embedded into a large-scale metabolic network, resulting in flux values that are in good accordance with those derived from dynamic simulations.

Utilizing the presented sensitivity analysis in combination with a classical approach, using control coefficients, revealed several indications: (i) Classical analysis showed a strong influence of the hexose phosphate balance on overall dynamics. (ii) Both, classical and dynamic sensitivity analysis, demonstrate a more pronounced and discernible impact of temperature on the system’s sensitivity compared to genotype. Classical analysis shows notable differences between different mutants, by an increase of highly sensitive elements due to a lack of starch, but a decrease due to diminished carbon retrieval from starch or defects in flavonoid synthesis. (iii) The dynamic analysis revealed consistent patterns of response deviation from the wild type among mutants with diverse defects in central carbon metabolism, suggesting shared mechanisms to counteract or compensate for these deficiencies.

During model validation, deviations from the experimentally determined data were identified. The question is to what extent these deviations have an impact on the described indications. For the classical analysis, this is hard to evaluate, because the observed deviations do not allow for specific insight on a parameter level, because they are all interconnected through the model network. Still, since the model parameters, which are subject of the metabolic control analysis, were derived in an extensive and highly constrained process as described in the methods section, and the mentioned deviations only occur for sensitivity scores that are estimated from dynamics of a perturbed system, there is a solid basis for the indications of the classical metabolic control analysis. The further analyses, on the other hand, are based on the sensitivity scores. Fortunately, as the temporal analysis considers the changes of sensitivity scores over time, it is not relevant if these are always higher or lower for some metabolites, which would be the consequence of the deviations. However, the principal component analysis would directly be impacted. Therefore, we performed a robustness test by repeatedly applying noise that corresponds to the observed deviations before performing the PCA. Although the quantitative results of the PCA were changed as expected, the clear separation of day 0 data as depicted in Fig. 5D was conserved throughout all repetitions. This robustness test is further described in Supplementary Section 2 and exemplary results are illustrated in Supplementary Fig. 4.

These considerations and tests support the notion that the model is well suited for the used analyses, despite some deviations from sensitivity scores derived from the experimental data.

When looking at the model structure as shown in Fig. 1, at first glance, it seems obvious that *f*, the parameter responsible for F6P-G6P balancing in connection to the PGI reaction, can have a strong impact on carbon partitioning, since it defines the fraction of carbon that can flow into the lower right part of the scheme (G6P, Suc, Starch, Glc and Frc) or into the upper left part (F6P, anthocyanins and other pathways), representing primary and secondary metabolism and carbon recycling, respectively. This does not mean it is used as a main regulator of carbon partitioning in vivo, but from a modeling point of view, its value has a strong influence on the overall model behavior. Nevertheless, this relationship needs to be balanced because F6P and G6P are both needed for an adequate flux through the rSPS reaction. Interestingly, when looking at the control coefficients of *f* for each system measure, there is a strong positive effect on Glc concentration, but an equally strong negative effect on Frc concentration, which is not immediately obvious by intuition. Here, the negative feedback of G6P plays an important role. Because the F6P/G6P balance emphasizes G6P, but the flux through rSPS is limited, there is an accumulation of G6P, that in turn blocks the activity of glucokinase via negative feedback, leading to an accumulation of Glc as well. This accumulation of Glc in turn, inhibits invertase activity and consequently leads to a depletion of Frc.

Given the high control coefficients of *f*, the rPGI reaction appears to be the most prominent control point for carbon distribution in the system. However, one needs to pay attention to the fact that the presented model is considerably simplified and condensed and the considered rPGI reaction comprises both, plastid and cytosol reactions. Compartmentalization and subcellular organization has been pointed out to play an important role for the central carbon metabolism and in terms of cold acclimation^37,38^. It is an appropriate assumption that not only the ratio of hexose phosphates, but also their subcellular proportions are of importance for the coordination of carbon partitioning. An extension of this study could focus on exploring this aspect. Nonetheless, these observations underline the critical importance of F6P/G6P balancing and a fine-tuned interplay of related reactions and processes. This notion agrees with the indications in Kitashova et al. 2022, as the rSPS reaction directly affects the balance of these hexoses.

Additionally, the new experimental data contains interesting insight connected to F6P/G6P balancing, by revealing that, for starch related mutants, the qualitative changes of F6P concentrations under high light and after acclimation are much closer to those of the wild type, than those of flavonoid related mutants. They increase for longer exposure while those of flavonoid related mutants decrease. With G6P, it is the other way around. For the wild type and flavonoid related mutants G6P concentrations were found to be stable, while those of starch related mutants decrease. Since flavonoid synthesis depends on F6P and starch synthesis depends on G6P, these qualitative deviations from wild type behavior directly indicate the affected parts of central carbon metabolism for each category of mutants.

Although (ii), the more pronounced and discernible impact of temperature, and (iii), the consistent differences to Col-0 in response patterns, are two separate observations, they fit into a coherent picture. Considering a more exposed alteration of the systems sensitivity through a temperature shift compared to the metabolic defects, suggests a successful compensation mechanism for the different mutants and it has been shown that there are mechanisms that can compensate gene knockouts in *Arabidopsis thaliana*^39^. Shared, or partially shared, compensation mechanisms for different deficiencies would be beneficial for the plant, because it would not require a specifically tailored response for all different kinds of limitations, enabling a more economical use of resources. The observed alterations in response patterns hint to a general or shared mechanism like this because the observed patterns are always of the same quality. If different compensation programs were at play, one would expect more apparent differences between the responses, at least between starch related and flavonoid related mutations, like more pronounced or opposing sensitivity changes for some reactions or metabolites. It has been observed that most metabolic heat shock responses are also responses to cold shock^11^, demonstrating the existence of common stress response mechanisms in the context of central carbon metabolism.

Even though these similarities are remarkable there are noticeable differences. When looking at the temporal evolution of the aggregated sensitivities in Fig. 6, we see that the starch related mutants show the highest deflection and overall deviation from Col-0. Also, the aggregated sensitivity of Col-0 increases up until day 14. While the time courses of the flavonoid related mutants seem to saturate, the values of the starch related mutants drastically decrease again between day 7 and 14, indicating that this enhanced sensitivity cannot be maintained throughout the acclimation process by these mutants. One can interpret this observation in the sense that defects in the primary metabolism, especially starch, might be more severe for successful acclimation, highlighting the central role of starch within this process.

The deviations from the Col-0 sensitivity scores seem not to affect the already sensitive parts of the system around Frc and Glc, even though we see differences in the number of highly sensitive elements between different genotypes. This might be a safety feature of the compensation mechanism, to make sure that these sensitive vital parts are maintained against destabilization. Although, a substantial difference in subcellular reprogramming of sucrose cleavage by invertase between natural genotypes of *A. thaliana* has been demonstrated with model simulations^40^, this is not necessarily the case between mutant genotypes that are not specialized to grow in different habitats. The high control coefficients for Frc, Glc and neighboring reactions support the concept of a buffering effect of futile cycling against external perturbations^21,41–46^, since it provides favorable conditions for a fine-tuned and flexible system. The fructokinase and glucokinase reactions also have a high sensitivity to changes in photosynthesis rates. Like this, the system can react fast and reliably to a sudden imbalance of sugars. The multiple feedback inhibitions provide a good backup, preventing overcompensation.

It is worth noticing that on day 0 (before acclimation), there is a significant quantitative difference between the deviations of the flavonol related and the starch related mutants for the rPGI and rSPS reactions. The *γ*_x_^nps^ values of the starch mutants are much closer to Col-0, while the absolute values of the flavonoid mutants are generally lower for these reactions. It might be the case that under normal conditions, these mutants naturally divert less carbon into the secondary pathways, since these are partially defective, so there is more carbon available that can potentially feed primary metabolism, rendering the initial steps less sensitive. This is in accordance with indications from the calibration, where the parameters k_ANT_ and k_r2_ have the lowest values for the two flavonol mutants.

Similar compensation programs, as indicated by the similar response patterns, for mutations in different metabolic branches hint to bi- or unidirectional signaling between these branches. Previous findings suggested a unidirectional signaling link between starch and flavonoid metabolism^25^ which supports the findings of the present study.

A long-term objective for further investigation is the identification of potential common compensation mechanisms. It could be part of an already studied stress response. Additionally, the more downstream effects of the investigated mutations in combination with cold stress could be examined to see if and at which level the differences become more specific.

## Methods

### Model equations

The presented model uses a total of 5 differential equations based on 11 corresponding rate equations.

$$\begin{array}{lll}\frac{{dF}6P}{{dt}} \; =\;{rNPS}-{rSPS}-{rPG}{I}_{1}-{rANTHO}-r2+{rFRCK}\\ \frac{{dG}6P}{{dt}} \; =\;{rPG}{I}_{2}-{rSPS}-{rST}{A}_{{syn}}+{rGLCK}\\ \frac{{dSuc}}{{dt}} \; =\;{rSPS}-r2-{rINV}\\ \frac{{dFrc}}{{dt}} \; =\;{rINV}-{rFRCK}\\ \frac{{dGlc}}{{dt}} \; =\;{rINV}-{rGLCK}+{rST}{A}_{deg }\end{array}$$

**Equation System 1**: Differential Equations

$$\begin{array}{lll}{rNPS} & =&\frac{1}{6}\cdot {k}_{{NPS}}\\ {rSPS} & =&v_{\max }^{{SPS}}\cdot F6P\cdot {k}_{{udp}}\cdot G6P\\ &&\cdot {\left(K{m}_{a}^{{SPS}}\cdot F6P+\left(K{m}_{b}^{{SPS}}+F6P\right)\cdot {k}_{{udp}}\cdot G6P+{K}_{i}^{{SPS}}\cdot K{m}_{a}^{{SPS}}\right)}^{-1}\\ {rFRCK} & =&{v}_{\max }^{{FRCK}}\cdot \frac{{Frc}}{\left(K{m}^{{FRCK}}+{Frc}\right)\cdot \left(1+\frac{F6P}{K{i}^{{FRCK}}}\right)}\\ {rGLCK} & =&{v}_{\max }^{{GLCK}}\cdot \frac{{Glc}}{\left(K{m}^{{GLCK}}+{Glc}\right)\cdot \left(1+\frac{G6P}{K{i}^{{GLCK}}}\right)}\\ {rINV} & =&{v}_{\max }^{{INV}}\cdot \frac{{Suc}}{\left(K{m}^{{INV}}\cdot \left(1+\frac{{Frc}}{K{i}^{{INV}1}}\right)+{Suc}\right)\cdot \left(1+\frac{{Glc}}{K{i}^{{INV}2}}\right)}\\ {rPG}{I}_{1} & =&{v}_{\max }^{{PGI}}\cdot \frac{F6P}{K{m}^{{PGI}}\cdot \left(1+\frac{G6P}{K{i}^{{PGI}}}\right)+F6P}\cdot f\\ {rPG}{I}_{2} & =&{v}_{\max }^{{PGI}}\cdot \frac{G6P}{K{m}^{{PGI}}\cdot \left(1+\frac{F6P}{K{i}^{{PGI}}}\right)+G6P}\cdot \left(1-f\right)\\ r1 & =&{k}_{1}^{\exp }\cdot {Suc}\\ r2 &=& {k}_{2}^{\exp }\cdot F6P\\ {\rm{rST}}{A}_{{syn}} &=& {k}_{{STA}}\cdot G6P\\ {rANTHO} & =&{k}_{{ANT}}\cdot F6P\end{array}$$

**Equation System 2:** Rate Equations

### Parameter re-estimation

For this study, we refined the initial model and re-estimated the parameters using the experimental data provided by Kitashova et al. (2022). The experimental data presented in this study was solely used for validation purposes as described in the model validation section.

Matching the composition of data presented in Kitashova et al. 2022, the model parameters were optimized to fulfill a steady state assumption for each genotype and time point pair, resulting in 25 conditional model realizations (parameterizations), representing different stages of acclimation for the different genotypes. While keeping the original structure of the model, here, we applied a different parametrization strategy by restricting all K_m_ and K_i_ values to common bounds for all genotypes and time points. Additionally, these bounds had to meet conditions derived from literature^32–36^:

- K_mFRCK_ > K_mGLCK_
- K_mINV_ similar or close to K_mFRCK_
- K_mFRCK_ > K_mPGI_ > K_mFRCK_

All corresponding estimated K_m_ and K_i_ values are within a range of one order of magnitude between each set. It is reasonable to assume the possibility of changes in K_m_ values over time, especially during a process of metabolic rearrangement like acclimation due to, for example, post translational modifications such as phosphorylation^47–49^.

This way, we were able to identify a new refined set of parameters for each combination of genotype and time point, sharing a common, close range of K_m_ and K_i_ values. In the context of parameterization, it is also important to mention that, while the model considers starch degradation, the degradation rate was set to 0 for the 25 conditional parameterizations, since they represent steady state conditions during midday and most starch degradation usually happens during the night under ambient temperature / control condition. But, as the starch production rates are estimated in a net balance over several days as described in Kitashova et al. 2022, relative changes of mean starch production and degradation are summarized in the production rate parameter *k*_*STA*_ and no explicit rate equation is defined for *rSTAdeg*. The model with the systematically revised parameters was then subjected to a sensitivity analysis, as described in the results section.

### Simulations and analyses

For all simulations the free software tool Tellurium^50^ (version 2.2.7) was used, utilizing the CVODE integrator to solve the set of ODEs. To perform metabolic control analysis, methods of libroadrunner^51,52^ within Tellurium were used. The free Matlab software package Data2Dynamics^53^ was used for parameter estimation.

Analysis of variance (ANOVA) was performed in R/RStudio^54^. Here, we conducted a two-way factorial ANOVA (illustrated in Fig. 2) to determine significant differences in hexose phosphate dynamics under cold and high light. For this analysis, metabolites were treated as dependent variables and genotype and time points as categorical factors. The interaction between genotype and time point (i.e., genotype × time point) was included in the analysis, which was performed using the aov() function in R.

The principal component analysis in the obervations and insights section was performed using scikit learn’s PCA method from the decomposition package^55^. This method utilizes singular value decomposition to project the data to a lower dimensional space. Before performing the PCA, the data was standardized using scikit-learn’s StandardScaler from the preprocessing package^55^. This scaler removes the dataset mean from each value and scales the result by the dataset standard deviation.

### Experimental procedures

*Arabidopsis thaliana* accession Col-0 and homozygous T-DNA insertion lines *bam3* (beta amylase 3, line SALK_041214, locus AT4G17090), *chs* (chalcone synthase, line SALK_020583C, locus AT5G13930), *f3h* (flavanone 3-hydroxylase, line SALK_113904C, locus AT3G51240), as well as the SNP mutant *pgm1* (plastidial phosphoglucomutase, TAIR stock CS3092, locus AT5G51820) were grown on a 1:1 mixture of GS90 soil and vermiculite in a climate chamber under controlled conditions (8 h/16 h day/night; 100 µmol m^−2^ s^−1^ ; 22 °C/16 °C; 60% relative humidity). Following two weeks of further growth under long day conditions (16 h/8 h day/night; 100 µmol m^−2^ s^−1^ ; 22 °C/16 °C), plants were shifted to 4 °C (16 h/8 h day/night; 90–100 µmol m^−2^ s^−1^ ; 4 °C/4 °C). On the 14th day of cold, plants were either (i) sampled after 3 h of ambient light; or (ii) exposed to elevated light (240–270 µmol m^−2^ s^−1^ ; 4 °C/4 °C) and sampled after 3 h and 6 h of elevated light. Each sample consisted of 1 leaf rosette which was snap-frozen in liquid nitrogen, ground to a fine powder and freeze-dried.

Fructose 6-phosphate (F6P) and glucose 6-phosphate (G6P) amounts were quantified as described before^56^. In brief, F6P and G6P were extracted with 16% (w/v) trichloroacetic acid in diethyl ether and washed with 16% (w/v) trichloroacetic acid in 5 mM EGTA. F6P and G6P amounts were determined in a 2-step cycling assay, catalyzing equimolar interconversion of hexose-phosphates into NADPH + H^+^, which was photometrically determined in a reaction with thiazolyl blue and phenazine methosulfate at 570 nm.

## Supplementary information

Supplementary information

## Data Availability

The full, raw set of experimental data measured for this study is provided in Supplementary Table 1. The experimental data used to re-parameterize the model in provided in the Supporting Information of the referenced study Kitashova et al. 2022^25^ (10.1111/pce.14483). The full set of model parameters used for the presented simulations and analyses are provided in Supplementary Table 2.
