# Supplementary material for: Plant cold acclimation and its impact on sensitivity of carbohydrate metabolism
Source: NPJ Syst Biol Appl. 2025 Mar 19;11:28. doi: 10.1038/s41540-025-00505-1 (PMC11923053; doi:10.1038/s41540-025-00505-1)
Supplement: Supplementary file 1 — Supplementary information [file 41540_2025_505_MOESM1_ESM.pdf]

Plant cold acclimation and its impact on sensitivity of carbohydrate metabolism

Stephan O. Adler[1], Anastasia Kitashova[2], Ana Bulović[1], Thomas Nägele[2], Edda Klipp[1]*

[1] Theoretical Biophysics, Institute of Biology, Humboldt-Universität zu Berlin, Berlin, Germany.
[2] Plant Evolutionary Cell Biology, Faculty of Biology, Ludwig-Maximilians-Universität München, Planegg-Martinsried, Germany.
* Corresponding Author, edda.klipp@rz.hu-berlin.de

Supplementary Material

Supplementary Section 1 – Metabolic Control Analysis

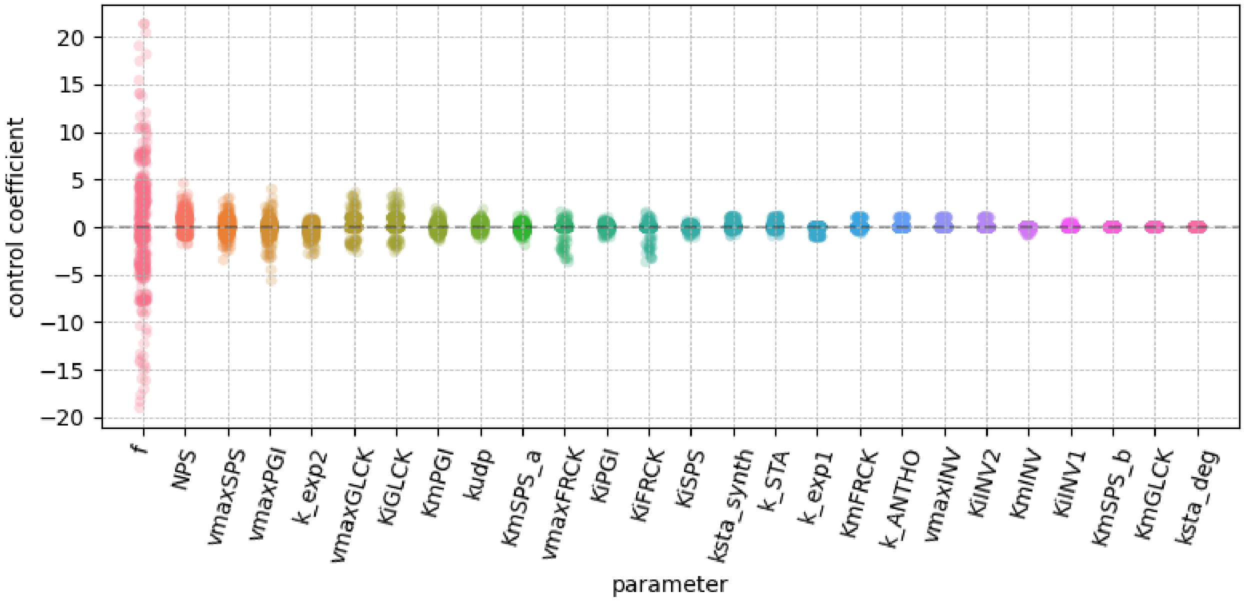

Supplementary Figure 1: Control coefficient overview of all model parameters. Each dot represents the control coefficient of the respective parameter on either a flux or a concentration, for one genotype-day combination. This results in 5*5*15 = 375 control coefficients per parameter (genotypes * days * (fluxes + components)). The colors have no particular meaning and serve a better visual distinction between the columns.

For this analysis the control coefficients of various model parameters and fluxes were estimated and compared. These control coefficients quantify the relative change of a flux or concentration when another flux, concentration or a parameter is changed.

The equations read:

$$C_p^X = \frac{\mathrm{d}\, ln\, X}{\mathrm{d}\, ln\, p}$$

Where C is the control coefficient, X is the concentration or flux of interest and p is the parameter. Supplementary Figure 1 clearly illustrates the strong influence of parameter *f* on the model components, compared to all other model parameters.

When looking at the control coefficients of *f* for each flux and concentration individually, one can notice a strong influence on Glc and Frc, which are both not directly connected to the *f*-dependent reactions. Also the influence on most other measures is relatively high compared to control coefficients of other parameters. It ranges to values of +/- 8 to 15 for at least one genotype-day combination, except for rNPS, which is completely independent.

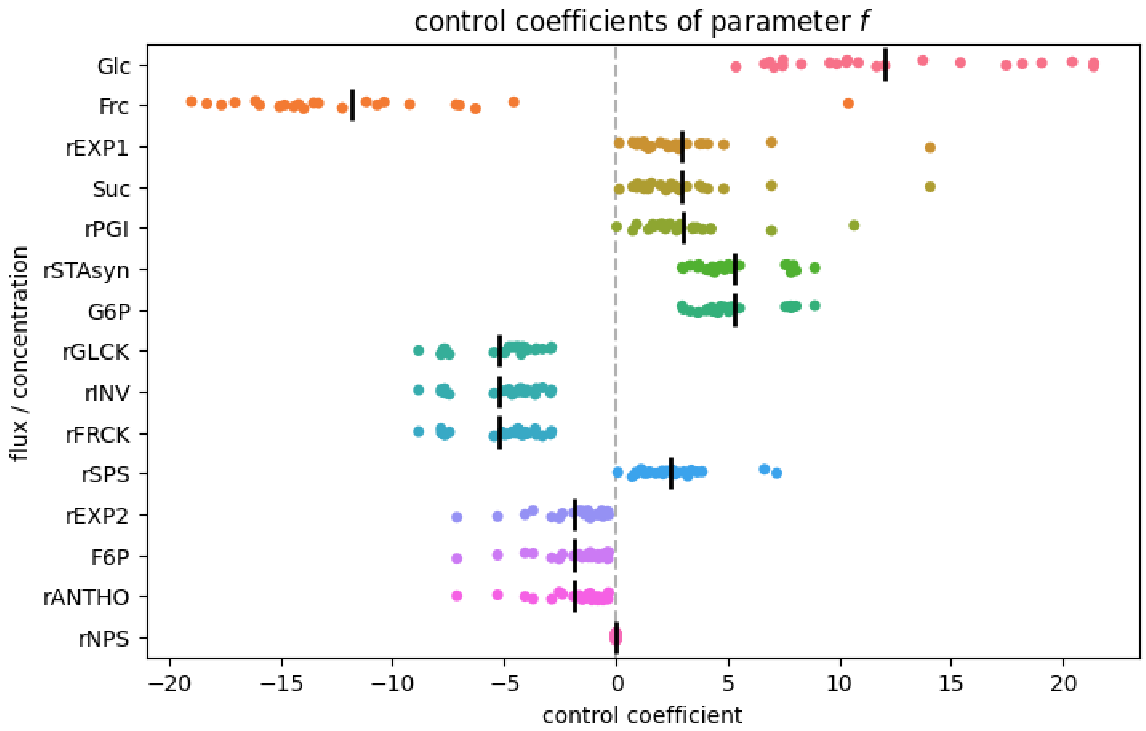

Supplementary Figure 2: Control coefficients of parameter *f*. Each dot represents the control coefficient of *f* on the respective flux or concentration, for one genotype-day combination. This results in 5*5 = 25 control coefficients per measure (genotypes * days). The colors have no particular meaning and serve a better visual distinction between the columns.

Supplementary Section 2 – Principal component analysis

This section shows more results of the principal component analysis described in 2.4. Here, also the results of PC3 and PC4 are shown in Supplementary Figure 3. One can notice that PC3 and PC4 each show a smaller spread along their axes, compared to PC1 and PC2. Also, when looking at the areas spanned by the respective values for each mutant or day, no particular pattern, like the clear separation of day 0 for PC1, can be observed.

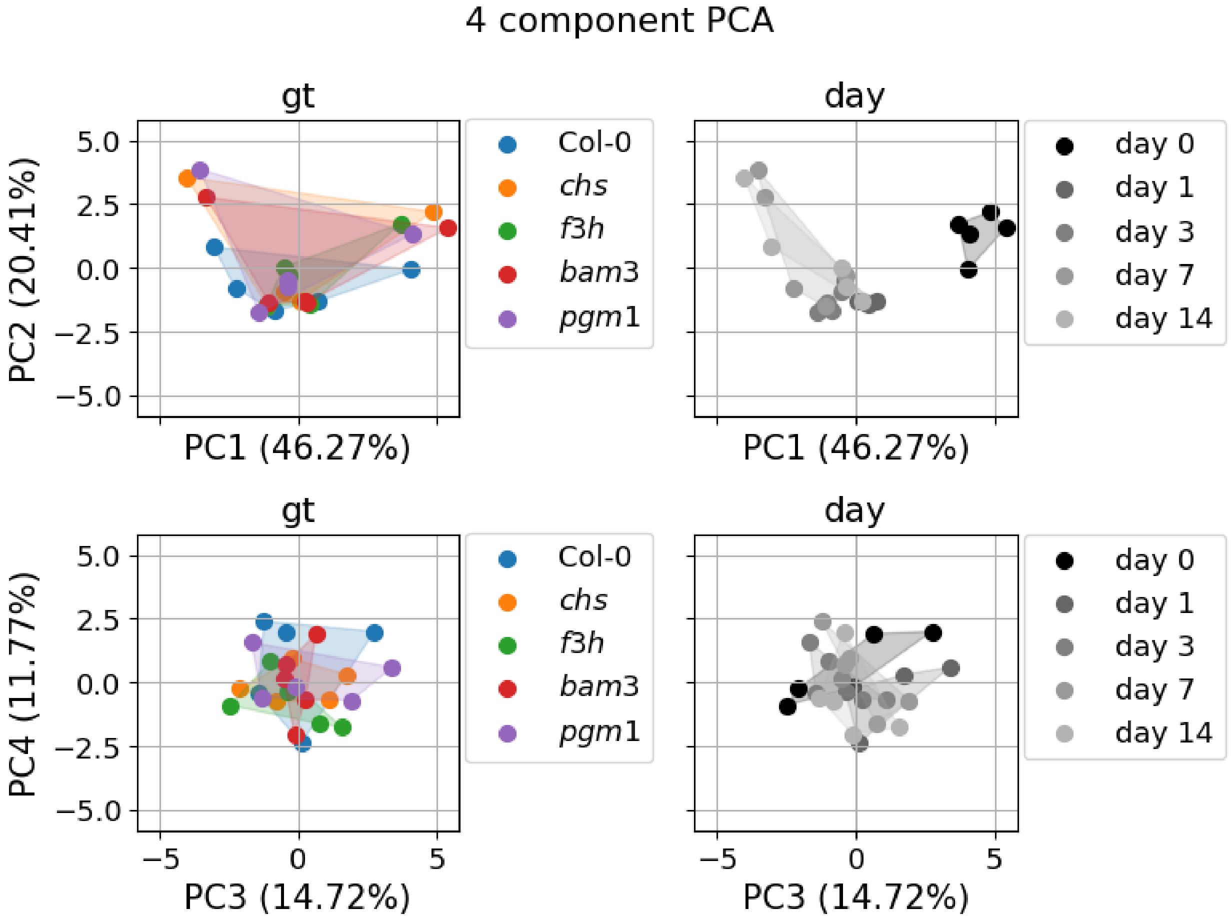

Supplementary Figure 3: Four-component PCA. All panels show the results of a principal component analysis for variations in γ_x^{nps}. The upper two panels show PC1 against PC2, while the lower two panels show PC3 against PC4. The respective contribution of each component is given in % in the axis labels. In the left two panels, the data points of each mutant span an area and are color coded accordingly. The data points in the right two panels are the same, but span areas according to their days. Here, the corresponding grey scale saturation decreases for later days.

To account for the fact that there are deviations between sensitivity scores derived from experimental and simulation data, a robustness analysis was performed to test, if the observations from the PCA still show if the observed deviations are applied to the data. The procedure of this analysis is explained in the figure caption.

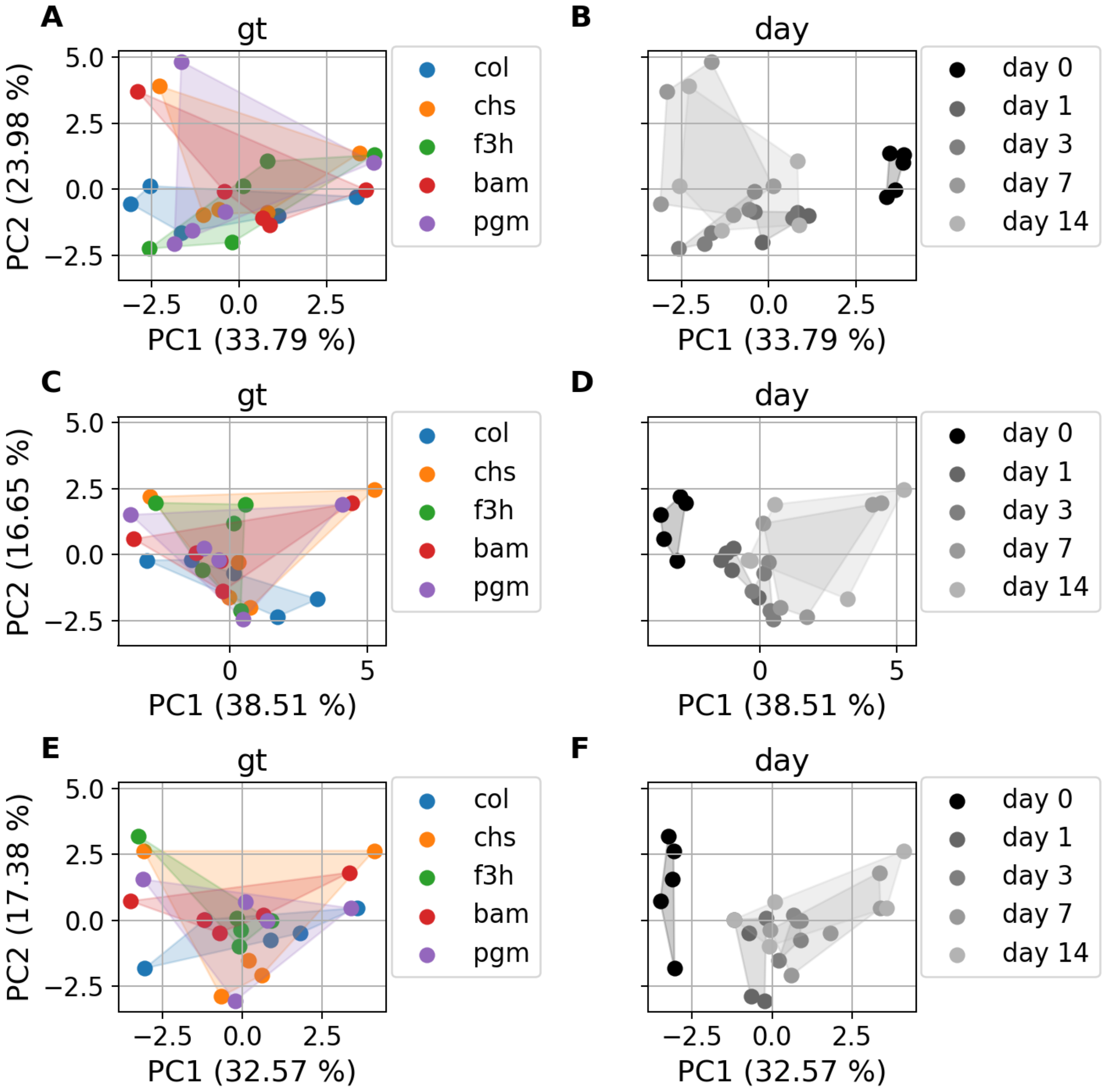

Supplementary Figure 4: PCA with artificial noise. The three panels (A/B, C/D and E/F) each show the results of a PCA for variations in γ_x^{nps}, as shown in Figure 5 of the main text. The difference here is that, for each panel, artificial noise was added to the computed sensitivity scores before performing the PCA. Based on the comparison of experimental and simulation data in Figure 3, the relative deviations ($\gamma_x^{nps,\, data}$ / $\gamma_x^{nps,\, sim}$) were extracted and randomly multiplied to all sensitivity scores. Here, each of the 10 deviation values had an equal probability to be assigned to each of the γ_x^{nps} values.

As can be seen in the figure, in all the panels, day 0 data is separated from data of all other days, while the genotypes show no obvious separation. This behavior was tested and seen for at least 30 random noise applications as described. Three are given here, as examples.
This underlines the robustness of the indication that the temperature change from 22/16 °C to 4 °C has a stronger influence on sensitivity towards changes in nps rates than the genotypes have.
The fact that the region occupied by day 0 data switches sides for different noise applications is not surprising, because the noise changes the composition and explained variance of the components, as indicated in the axis labels.

Supplementary Section 3 – Experimental Data

sample number	Genotype	time in high light	G6P	F6P
1	Col-0	0h	11.18	2.16
2	Col-0	0h	20.71	8.19
3	Col-0	0h	13.77	
4	Col-0	0h	6.50	3.29
5	Col-0	0h	21.82	1.37
6	Col-0	0h	5.49	1.08
7	Col-0	3h	5.96	1.32
8	Col-0	3h	11.96	3.12
9	Col-0	3h	6.01	7.79
10	Col-0	3h	13.15	1.59
11	Col-0	3h	16.94	3.95
12	Col-0	3h	8.42	2.72
13	Col-0	6h	7.41	8.51
14	Col-0	6h	13.03	1.35
15	Col-0	6h	14.24	0.76
16	Col-0	6h	10.61	5.76
17	Col-0	6h	14.33	1.46
18	Col-0	6h	5.95	8.82
19	bam3	0h	14.47	6.03
20	bam3	0h	15.79	3.21
21	bam3	0h	3.82	1.44
22	bam3	0h	4.62	1.32
23	bam3	0h	18.11	3.14
24	bam3	0h	11.27	2.88
25	bam3	3h	11.25	1.58
26	bam3	3h	16.22	12.41
27	bam3	3h	5.66	5.85
28	bam3	3h	9.57	3.51
29	bam3	3h	11.91	0.55
30	bam3	3h	3.57	0.73
31	bam3	6h	8.65	8.65
32	bam3	6h	3.53	2.72
33	bam3	6h	6.30	3.14
34	bam3	6h	6.99	4.13
35	bam3	6h	16.72	1.07
36	bam3	6h	6.53	4.07
37	chs	0h	1.12	0.67
38	chs	0h	12.70	7.85
39	chs	0h	12.59	42.26
40	chs	0h	11.48	1.71
41	chs	0h	10.83	3.24
42	chs	0h	11.70	
43	chs	3h	7.04	10.20
44	chs	3h	13.29	5.02
45	chs	3h	12.42	6.62
46	chs	3h	12.44	3.82
49	chs	6h	9.87	4.85
50	chs	6h	24.47	5.91
51	chs	6h	11.13	3.82
52	chs	6h	15.85	
53	chs	6h	13.88	5.07
54	chs	6h	9.92	6.99
55	f3h	0h	12.96	12.62
56	f3h	0h	14.36	2.37
57	f3h	0h	10.38	35.10
58	f3h	0h	9.29	0.29
59	f3h	0h	14.30	3.88
60	f3h	0h	8.96	3.89
61	f3h	3h	11.70	3.53
62	f3h	3h	13.52	4.14
63	f3h	3h	21.78	
64	f3h	3h	9.34	1.16
65	f3h	3h	14.00	2.34
66	f3h	3h	6.11	8.23
67	f3h	6h	22.10	9.20
68	f3h	6h	10.55	1.82
69	f3h	6h	10.75	1.03
70	f3h	6h	17.62	9.61
71	f3h	6h	15.71	1.83
72	f3h	6h	8.94	0.90
73	pgm1	0h	27.26	2.44
74	pgm1	0h	24.88	8.92
75	pgm1	0h	32.28	9.94
76	pgm1	0h	23.17	0.36
77	pgm1	0h	27.93	1.16
78	pgm1	0h	5.66	0.64
79	pgm1	3h	29.91	6.75
80	pgm1	3h	36.21	16.64
81	pgm1	3h	45.56	2.10
82	pgm1	3h	24.22	1.23
83	pgm1	3h	23.61	3.37
84	pgm1	3h	21.77	
85	pgm1	6h	20.49	25.89
86	pgm1	6h	28.30	6.63
87	pgm1	6h	16.42	0.89
88	pgm1	6h	14.94	7.10
89	pgm1	6h	25.91	5.67
90	pgm1	6h	9.82	0.77

Supplementary Table 1: Experimental Data. The table shows all measured Data as described in 4.4 and 2.2. The concentrations of G6P and F6P are provided in µmol / gDW.

Supplementary Section 4 – Model Parameters

		Genotype						
**day**	**parameter**	**Col-0**	**chs**	**f3h**	**bam3**	**pgm1**	**dimension**	**log**
0	KiFRCK	-0.90	-0.75	-1.13	-0.94	-1.16	µmol / gDW	1
	KiGLCK	-1.23	-1.44	-1.74	-1.12	-1.66	µmol / gDW	1
	KiINV1	1.37	0.90	0.97	1.29	0.95	µmol / gDW	1
	KiINV2	-1.02	-1.17	-1.44	-1.04	-1.36	µmol / gDW	1
	KiPGI	0.61	0.75	0.50	0.10	0.79	µmol / gDW	1
	KiSPS	0.90	0.40	0.49	0.71	0.62	µmol / gDW	1
	KmFRCK	1.64	1.53	1.80	1.64	1.86	µmol / gDW	1
	KmGLCK	-2.85	-2.77	-2.46	-3.13	-2.69	µmol / gDW	1
	KmINV	1.69	1.79	2.20	1.74	1.89	µmol / gDW	1
	KmPGI	-0.60	-0.33	-0.42	-0.18	-0.61	µmol / gDW	1
	KmaSPS	0.42	-0.10	0.02	0.13	0.15	µmol / gDW	1
	KmbSPS	-1.40	-1.90	-1.79	-1.86	-1.71	µmol / gDW	1
	fPGI	0.54	0.60	0.57	0.73	0.56	dimensionless	0
	kANT	0.09	0.03	0.03	0.17	0.08	1 / atu	0
	kNPS	2.92	2.94	3.14	3.08	3.16	µmol / atu	1
	kSTA	1.25	0.67	0.62	2.45	0.00	1 / atu	0
	kexp1	0.07	1.74	1.42	0.18	0.44	1 / atu	0
	kexp2	111.49	37.84	88.09	301.03	182.08	1 / atu	0
	kudp	0.47	0.50	0.45	0.39	0.43	1 / atu	0
	vmaxFRCK	1.68	1.71	1.80	1.63	1.73	µmol / atu	1
	vmaxGLCK	1.52	1.70	1.49	1.45	1.69	µmol / atu	1
	vmaxINV	2.98	3.16	3.20	3.19	3.26	µmol / atu	1
	vmaxPGI	2.64	2.75	2.74	2.68	2.76	µmol / atu	1
	vmaxSPS	1.80	2.07	2.02	2.00	2.02	µmol / atu	1
1	KiFRCK	-1.40	-1.02	-1.12	-1.10	-1.15	µmol / gDW	1
	KiGLCK	-1.23	-1.14	-1.02	-1.01	-1.39	µmol / gDW	1
	KiINV1	1.86	1.60	1.59	1.68	1.34	µmol / gDW	1
	KiINV2	-0.79	-0.79	-0.81	-0.69	-1.06	µmol / gDW	1
	KiPGI	0.70	0.58	0.51	0.15	0.50	µmol / gDW	1
	KiSPS	0.40	0.40	0.81	0.40	0.40	µmol / gDW	1
	KmFRCK	2.10	1.72	1.82	1.80	2.10	µmol / gDW	1
	KmGLCK	-2.97	-3.06	-3.29	-3.17	-2.81	µmol / gDW	1
	KmINV	1.20	1.50	1.51	1.39	1.76	µmol / gDW	1
	KmPGI	-0.47	-0.51	-0.19	-0.30	-0.50	µmol / gDW	1
	KmaSPS	-0.10	-0.10	0.31	-0.10	-0.10	µmol / gDW	1
	KmbSPS	-1.90	-1.63	-1.41	-1.90	-1.90	µmol / gDW	1
	fPGI	0.59	0.58	0.65	0.67	0.65	dimensionless	0
	kANT	0.02	0.00	0.00	0.01	0.00	1 / atu	0
	kNPS	2.71	2.75	2.70	2.81	2.85	µmol / atu	1
	kSTA	-0.46	-0.10	-0.26	1.22	0.00	1 / atu	0
	kexp1	0.06	0.12	0.06	0.09	0.24	1 / atu	0
	kexp2	45.24	17.59	35.22	40.09	10.14	1 / atu	0
	kudp	0.50	0.50	0.29	0.49	0.50	1 / atu	0
	vmaxFRCK	1.06	1.00	1.08	1.22	1.20	µmol / atu	1
	vmaxGLCK	1.03	1.06	1.17	1.09	1.15	µmol / atu	1
	vmaxINV	2.64	2.72	2.79	2.59	2.74	µmol / atu	1
	vmaxPGI	1.98	1.98	1.79	1.99	1.93	µmol / atu	1
	vmaxSPS	1.19	1.24	1.43	1.15	1.42	µmol / atu	1

		Genotype						
**day**	**parameter**	**Col-0**	**chs**	**f3h**	**bam3**	**pgm1**	**dimension**	**log**
3	KiFRCK	-1.40	-1.36	-1.34	-1.40	-1.14	µmol / gDW	1
	KiGLCK	-1.19	-1.40	-1.22	-1.25	-1.31	µmol / gDW	1
	KiINV1	1.78	1.78	1.70	1.73	1.60	µmol / gDW	1
	KiINV2	-0.61	-0.57	-0.72	-0.66	-0.74	µmol / gDW	1
	KiPGI	0.52	0.39	0.60	0.74	0.81	µmol / gDW	1
	KiSPS	0.70	0.40	0.67	0.57	1.00	µmol / gDW	1
	KmFRCK	2.10	2.05	2.04	2.10	1.84	µmol / gDW	1
	KmGLCK	-3.40	-2.81	-3.03	-2.81	-2.89	µmol / gDW	1
	KmINV	1.32	1.27	1.39	1.37	1.50	µmol / gDW	1
	KmPGI	-0.52	-0.32	-0.72	-0.47	-0.50	µmol / gDW	1
	KmaSPS	0.19	-0.10	0.17	0.07	0.50	µmol / gDW	1
	KmbSPS	-1.59	-1.90	-1.63	-1.73	-1.29	µmol / gDW	1
	fPGI	0.62	0.60	0.61	0.62	0.56	dimensionless	0
	kANT	0.03	0.00	0.00	0.01	0.01	1 / atu	0
	kNPS	2.46	2.54	2.54	2.43	2.60	µmol / atu	1
	kSTA	1.33	1.12	0.85	1.64	0.00	1 / atu	0
	kexp1	0.02	0.05	0.04	0.02	0.04	1 / atu	0
	kexp2	17.27	10.16	16.73	12.01	7.47	1 / atu	0
	kudp	0.37	0.50	0.37	0.41	0.20	1 / atu	0
	vmaxFRCK	0.95	1.13	0.86	1.06	1.09	µmol / atu	1
	vmaxGLCK	0.83	1.07	0.91	0.94	1.19	µmol / atu	1
	vmaxINV	2.61	2.49	2.58	2.54	2.54	µmol / atu	1
	vmaxPGI	1.79	1.99	1.88	1.86	1.84	µmol / atu	1
	vmaxSPS	1.21	1.23	1.25	1.07	1.55	µmol / atu	1
7	KiFRCK	-1.27	-1.50	-1.50	-1.31	-1.39	µmol / gDW	1
	KiGLCK	-1.03	-1.16	-1.07	-1.25	-1.03	µmol / gDW	1
	KiINV1	1.89	1.92	1.92	1.45	1.74	µmol / gDW	1
	KiINV2	-0.50	-0.48	-0.48	-0.91	-0.63	µmol / gDW	1
	KiPGI	0.39	0.94	0.45	0.53	0.73	µmol / gDW	1
	KiSPS	0.40	0.40	0.30	0.40	0.41	µmol / gDW	1
	KmFRCK	1.97	2.20	2.20	2.02	2.09	µmol / gDW	1
	KmGLCK	-3.17	-3.01	-3.40	-2.95	-3.40	µmol / gDW	1
	KmINV	1.20	1.18	1.18	1.63	1.33	µmol / gDW	1
	KmPGI	-1.04	-0.63	-0.88	-0.82	-0.11	µmol / gDW	1
	KmaSPS	-0.10	-0.10	-0.20	-0.10	-0.05	µmol / gDW	1
	KmbSPS	-1.90	-1.90	-2.00	-1.85	-1.87	µmol / gDW	1
	fPGI	0.69	0.67	0.67	0.66	0.63	dimensionless	0
	kANT	0.21	0.00	0.01	0.04	0.02	1 / atu	0
	kNPS	2.42	2.46	2.33	2.23	2.43	µmol / atu	1
	kSTA	1.04	0.73	0.46	0.60	0.00	1 / atu	0
	kexp1	0.06	0.04	0.07	0.13	0.14	1 / atu	0
	kexp2	22.61	25.67	16.23	3.07	1.69	1 / atu	0
	kudp	0.50	0.50	0.50	0.50	0.50	1 / atu	0
	vmaxFRCK	0.99	1.17	1.08	1.16	1.30	µmol / atu	1
	vmaxGLCK	0.99	1.07	1.16	0.88	0.88	µmol / atu	1
	vmaxINV	2.62	2.50	2.46	2.50	2.48	µmol / atu	1
	vmaxPGI	1.60	1.62	1.96	1.72	2.02	µmol / atu	1
	vmaxSPS	1.32	1.04	1.19	1.32	1.39	µmol / atu	1

		Genotype						
**day**	**parameter**	**Col-0**	**chs**	**f3h**	**bam3**	**pgm1**	**dimension**	**log**
14	KiFRCK	-1.11	-1.17	-1.34	-1.13	-1.22	µmol / gDW	1
	KiGLCK	-1.16	-1.29	-0.83	-1.30	-0.80	µmol / gDW	1
	KiINV1	1.56	1.69	1.58	1.56	1.33	µmol / gDW	1
	KiINV2	-0.84	-0.71	-0.82	-0.84	-0.79	µmol / gDW	1
	KiPGI	0.48	0.63	0.27	0.29	0.75	µmol / gDW	1
	KiSPS	0.40	0.49	0.40	0.42	0.63	µmol / gDW	1
	KmFRCK	1.81	1.88	2.04	1.83	2.10	µmol / gDW	1
	KmGLCK	-3.04	-2.91	-3.37	-2.90	-3.40	µmol / gDW	1
	KmINV	1.54	1.45	1.53	1.54	1.89	µmol / gDW	1
	KmPGI	-0.92	-0.59	-0.28	-0.59	-0.67	µmol / gDW	1
	KmaSPS	-0.10	-0.02	-0.10	-0.08	0.28	µmol / gDW	1
	KmbSPS	-1.90	-1.76	-1.90	-1.88	-1.57	µmol / gDW	1
	fPGI	0.63	0.66	0.68	0.60	0.57	dimensionless	0
	kANT	-0.02	0.00	0.00	0.01	0.00	1 / atu	0
	kNPS	2.29	2.15	2.22	2.19	2.35	µmol / atu	1
	kSTA	-0.22	-0.08	-0.04	-0.11	0.00	1 / atu	0
	kexp1	0.09	0.08	0.06	0.04	0.08	1 / atu	0
	kexp2	5.89	0.14	4.08	9.87	7.10	1 / atu	0
	kudp	0.50	0.46	0.50	0.49	0.37	1 / atu	0
	vmaxFRCK	0.87	0.99	1.22	0.71	1.37	µmol / atu	1
	vmaxGLCK	0.95	1.18	0.69	0.99	0.95	µmol / atu	1
	vmaxINV	2.62	2.66	2.70	2.58	2.71	µmol / atu	1
	vmaxPGI	1.68	1.72	1.72	1.82	2.09	µmol / atu	1
	vmaxSPS	1.31	1.43	1.15	1.01	1.44	µmol / atu	1

Supplementary Table 2: Parameters. The table shows the used parameter values for all genotype-day combinations. The column “log” indicates that the values shown are simple numbers (0) or exponential factors (1). The abbreviation “atu” indicates “arbitrary time unit”.
